# Decoding Alzheimer’s disease: acetylcholine and dopamine pathway disruptions as early markers of cognitive decline

**DOI:** 10.1093/braincomms/fcaf057

**Published:** 2025-02-06

**Authors:** Claudio Zaccone, Annalisa Nobili, Marcello D’Amelio

**Affiliations:** Department of Medicine and Surgery, Università Campus Bio-Medico di Roma, 00128 Rome, Italy; Department of Medicine and Surgery, Università Campus Bio-Medico di Roma, 00128 Rome, Italy; Department of Experimental Neurosciences, IRCCS Santa Lucia Foundation, 00143 Rome, Italy; Department of Medicine and Surgery, Università Campus Bio-Medico di Roma, 00128 Rome, Italy; Department of Experimental Neurosciences, IRCCS Santa Lucia Foundation, 00143 Rome, Italy

## Abstract

This scientific commentary refers to ‘Changes in neurotransmitter-related functional connectivity along the Alzheimer’s disease continuum’, by Manca *et al*. (https://doi.org/10.1093/braincomms/fcaf008).

**This scientific commentary refers to ‘Changes in neurotransmitter-related functional connectivity along the Alzheimer’s disease continuum’, by Manca *et al*. (https://doi.org/10.1093/braincomms/fcaf008).**

Alzheimer’s disease is the most prevalent cause of dementia. Despite being identified over a century ago, its fundamental pathological mechanisms remain elusive. The ‘amyloidogenic hypothesis’, which is the dominant explanation for Alzheimer’s disease, attributes the disease to the neurotoxic effects of the self-aggregating amyloid β (Aβ) peptide. However, this hypothesis does not fully capture the complexity of Alzheimer’s disease pathogenesis, as Aβ plaques are also found in cognitively healthy elderly individuals.^1^ Additionally, new therapies targeting Aβ, in both its monomeric and aggregated forms, have shown limited success and are often associated with significant side effects.^2^

Recent research, derived by previous results obtained in Alzheimer’s disease mouse models,^3,4^ highlights the early involvement in Alzheimer’s disease of the nuclei within the isodendritic core, a group of specific neurons located in the brainstem and basal forebrain, that project to various central nervous system targets.^5^ The depletion of neurotransmitters in their projecting areas not only impairs the function of affecting regions but also reduces the neurotrophic and immunomodulatory roles of neurotransmitters on neurons and glial cells.^6-10^ This progressive decline may contribute to the appearance of cognitive and psychiatric deficits, depending on the brain regions impacted.

In this context, the study by Manca *et al*.,^11^ in this issue of *Brain Communications*, represents a significant contribution to the understanding of Alzheimer’s disease pathogenesis. By applying the innovative Receptor-Enriched Analysis of functional Connectivity by Targets (REACT), which combined PET atlases with resting state functional connectivity (FC) MRI, they examined variation in dopamine and acetylcholine functional connectivity across different brain regions. Their retrospective analysis included patients suffering from mild cognitive impairment due to Alzheimer’s disease (AD-MCI), patients with Alzheimer’s disease–related dementia (AD-dementia) and cognitively unimpaired controls (CU).

Manca *et al*. identified distinct patterns of dopamine and acetylcholine system alterations among the groups. Dopaminergic FC differences, assessed using PET atlases of the dopamine transporter (DAT) and the dopaminergic receptor D1, show variability across the Alzheimer’s disease continuum, suggesting stage-specific involvement in the dopaminergic pathway. For instance, reduced nigrostriatal DAT-related FC in the left superior temporal gyrus was present in AD-MCI patients but absent in AD-dementia. Conversely, lower mesocorticolimbic D1-related FC in the left praecuneus was observed in AD-dementia whereas higher mesocorticolimbic DAT-related FC in the bilateral thalamic nuclei was noted in AD dementia compared to CU individuals. These findings imply that dysfunction may manifest differently across disease stages and warrant further investigation.

Notably, no between-group differences or cognition-FC associations related to amyloid pathology were detected in the dopaminergic systems, suggesting that dopaminergic degeneration and Aβ accumulation may be independent processes occurring simultaneously.

In contrast, the cholinergic system exhibited more consistent disruptions across the Alzheimer’s disease spectrum. Both AD-MCI and AD-dementia groups showed reduced M1-related FC in the right superior/middle temporal gyri. Aβ-positive AD-dementia patients also display lower M1-related FC in the right posterior middle temporal gyrus compared to Aβ-negative CU individuals. Furthermore, the AD-MCI group exhibited decreased VAChT-related FC in the right postcentral and precentral gyri and lower α_4_β_2_-related FC in the right lingual gyrus and left posterior cingulate cortex, aligning with the early and pervasive involvement of the cholinergic system in Alzheimer’s disease and leading to diminished acetylcholine transmission.

Manca *et al*. also uncover intriguing associations in both the dopamine and acetylcholine systems. Higher M1-related FC in regions such as the right superior/middle temporal, right cerebellar (culmen) and bilateral posterior cingulate regions was linked to better cognitive score. Episodic memory function showed negative association with D1-related FC in frontal regions and DAT-related FC in the anterior cingulate cortex, while positive association was found with DAT-related FC in the praecuneus and posterior cingulate cortices. For the cholinergic system, positive correlations were identified between the Logical Memory test delayed-recall (LM-DR) scores and VAChT-related FC in sensory-motor areas, as well as M1-related FC in the parietal and temporal cortices. Interestingly, a positive correlation between LM-DR scores and M1-related FC in the fusiform gyrus and cerebellum was observed in Aβ-positive participants.

These findings underscore the complex and distinct role of dopaminergic and cholinergic systems in Alzheimer’s disease and highlight the potential of the REACT approach in elucidating the nuanced pathophysiology of the disease. Furthermore, these findings emphasized the heterogeneous nature of Alzheimer’s disease, in which neurotransmitter imbalances can vary across different stages and individuals, influenced by factors such as personal/genetic susceptibility and environmental conditions. The distinct vulnerability of specific nuclei within the isodendritic core likely contributes to this heterogeneity. Manca *et al*.’s research provides a valuable framework for future investigation and clinical trials exploring neurotransmitter dysregulation in Alzheimer’s disease, including its preclinical phase.

Although the study by Manca *et al*. has limitations, such as the lack of evaluation of other neurotransmitters and receptors, as well as the inconsistent assessment of Alzheimer’s disease biomarkers, their findings emphasize the potential for early Alzheimer’s disease diagnosis. Gaining insight into the cascade that leads to neurotransmitter imbalance could shed light on the early stages of the disease, paving the way for timely detection and intervention. This is particularly crucial as different neurotransmitter systems might be involved at the onset of the disease, opening the door for precision therapies tailored to individual Alzheimer’s disease patients. Moreover, as this study underscores the variability of neurotransmitter imbalances across the Alzheimer’s disease continuum, it becomes evident that a single medication effective for all stages of the disease will not be feasible.

In this context, future research could explore the progression of dysfunction across various neurotransmitter systems in familial Alzheimer’s disease, resolving discrepancies with previous findings.^12^ Mapping the timeline of neurodegeneration within this specific framework may offer insights applicable to sporadic forms of Alzheimer’s, ultimately guiding the development of more personalized diagnostic and therapeutic strategies.

## Data Availability

Data sharing is not applicable to this article as no new data were created or analysed in this study.
